# Society Meetings

**Published:** 1909-03

**Authors:** 


### SOCIETY MEETINGS.

---

THE Medical Society of the County of Erie, held its regular meeting Monday evening, February 15, 1909, at 8.15 o'clock, in the rooms of the Society of Natural Sciences, Buffalo Library Building. Program: L. Bradley Dorr, Silver Salts in Typhoid Fever; David Morrison, An Unusual Case in Obstetrics; John H. Grant, Whitlow or Felon; F. C. Busch, Practical Application of Research; Jacob S. Otto, Tendencies in Infant Feeding; Joseph Burke, Diagnosis of Pancreatic Affections.

---

THE Clinical Club held its regular meeting in the rooms of the Buffalo Society of Natural Sciences, Library building, Monday evening, February, 1, 1909, at 9 o'clock. The paper of the evening was entitled Antitoxins, Vaccins, and Bacterins with stereopticon slides, by Dr. Larned of Detroit.

---

THE Association of American Medical Colleges will hold its next annual meeting at the Academy of Medicine, 17 West 43d street, New York, March 15 and 16, 1909, under the presidency of Professor Eli H. Long, M. D., of the University of Buffalo. The Manhattan Hotel is the headquarters and Dr. Egbert Le Fevre, 52 West 56th street, New York, is chairman of the committee of arrangements.

---

THE Buffalo Medical Union held its 26th annual dinner at the Hotel Statler, Wednesday evening, February 3, 1906, with Dr. A. H. Briggs as toastmaster. At the table two seats were left vacant in memory of Dr. Grosvenor R. Trowbridge and Dr. Charles A. Ring who died since the last dinner. Dr. E. J. Meyer eulogised them in a brief but appropriate speech. Other toasts responded to were The Old Guard, by W. C. Phelps; Brotherly Love, by W. C. Krauss, and Before and After, by H. C. Buswell.

---

THE Roswell Park Medical Club, Buffalo, entertained the Physician's League, the woman's medical organisation, at a banquet given at The Markeen, Thursday evening, February 4, 1909.

---

THE Buffalo Academy of Medicine held meetings during the months of January and February, 1909, as follows:

*Section on Surgery*.—Tuesday evening, January 5. Program: Surgical Treatment of Chronic Arthritis, Edward H. Ochsner, Chicago, Ill. Discussion was opened by Dr. Bernard Bartow and Dr. Prescott Le Breton.

*Section on Medicine.*—Tuesday evening, January 12. Program: Symposium on tuberculosis. (1) An Outline of the Fight Against Tuberculosis in a city of the First-Class, Livingston Farrand, New York; (2) The Tuberculosis Problem in Buffalo, John H. Pryor; (3) Report of the Tuberculosis Day Camp, Geo. H. Eckel. There was also a demonstration, of the New Cutaneous Tests for Tuberculosis. Cases showing The Von Pirquet, The Moro and The Detre Reactions by Julius Ullman and Geo. H. Eckel.

*Section on Pathology.*—Saturday evening, January 23. Program: The General Principles of Percentage Feeding and the Laboratory Management of all Food Stuffs, Thomas Morgan Rotch, Professor of Pediatrics in the Harvard Medical School, Boston, Mass.

*Section on Obstetrics and Gynecology.*—Tuesday evening, January 26. Program: The Frequency, Etiology and Practical Significance of Funnel Shaped Pelves, J. Whitridge Williams, Baltimore, Md., Professor of Obstetrics, Johns Hopkins Medical School and author of Williams's Obstetrics.

*Section on Surgery.*—Tuesday evening, February 2. Program: The Diagnosis and Treatment of Vesical Tumors with Demonstration of an Operating Cystoscope, Hugh H. Young, Baltimore, Md., Professor of Genitourinary Surgery in the Johns Hopkins Medical School.

*Section of Medicine.*—Tuesday evening, February 9. Program: The Production of Clean Milk and its Behavior in the Digestive Tract, illustrated by stereopticon lantern slides, Frederick W. Howe, B. S., Boston, Mass., professor of physiological chemistry, food and dietetics at the State Normal School, Framingham, Mass. Discussion was opened by Irving M. Snow and Nelson G. Russell; Report of Three Cases of Pyloric Stenosis in Infants, Irving M. Snow.

*Section on Pathology.*—Tuesday evening, February 16. Program: The Type of Tubercle Bacilli which Produces the Different Kinds of Tuberculosis in New York, Wm. H. Park, Health Department, New York.

*Section on Obstetrics and Gynecology.*—Tuesday evening, February 23. Program: Salient Points of Normal Labor, Irving W. Potter; Salient Points of Abnormal Labor, Ludwig Schroeter; Practical use of Forceps, Kenneth McIlwraith, Professor of Obstetrics, University of Toronto; Some New Ideas Concerning the Care of the Eyes of the New Born, W. Linton Phillips.

The Rochester Academy of Medicine held meetings during the months of January and February at the Hotel Seneca, as follows:

*Section on Public Health.*—Wednesday, January 20. Program: Report of Cases Treated by Vaccine, Charles O. Boswell.

Wednesday, February 24: Influenza and Common Colds, John O. Roe.

*Section on Obstetrics, Gynecology, and Pediatrics.*—Friday, January 29. Program: The Ante-operative and Post-operative Care of Patients, N. G. Orchard. Discussion was opened by E. W. Mulligan.

Wednesday, February 17. Subinvolution of the Uterus, J. F. W. Whitbeck. Discussion was opened by O. E. Jones.

*Section on General Medicine.*—Wednesday, February 3. Program: The First Interview with the Patient, William S. Ely Focalised Epilepsy of Probable Arteriosclerotic Origin in a Boy of Thirteen, Edward L. Hanes.

*Section on Surgery.*—Wednesday, February 10. Program: Mechanical Lesions of the Sacro-iliac Joint, Ralph R. Fitch.

